# Long‐term patterns of breeding range expansion mirror invasion dynamics during recolonisation of a semi‐colonial raptor

**DOI:** 10.1111/1365-2656.70322

**Published:** 2026-08-12

**Authors:** Brittany C. Maxted, Richard A. Stillman, Phillipa K. Gillingham, Ross A. Hill, Tim Mackrill, Roy Dennis

**Affiliations:** ^1^ School of Life and Environmental Sciences Bournemouth University Poole UK; ^2^ Birds of Poole Harbour Wareham Dorset UK; ^3^ Roy Dennis Wildlife Foundation Forres Moray UK

**Keywords:** avian ecology, biogeography, hierarchical clustering, natal dispersal, nature recovery, population growth, raptor conservation, stratified diffusion

## Abstract

1. Range expansions are driven by a host of eco‐evolutionary mechanisms, with consequences for individual fitness, population dynamics and ecosystem function. Processes at the range front are particularly important in determining rates of expansion and resulting distributions.
2. Species recolonisations are a subset of range expansions which have become a global conservation priority, with a key goal to maximise the rate of spatial recovery. However, this is limited by a poor understanding of the underlying patterns expected during recolonisations and their mechanistic drivers.
3. We address this knowledge gap using a unique long‐term dataset, comprising nesting records across 54 years of recolonisation by Osprey *Pandion haliaetus* in Northern Scotland. We investigate spatiotemporal variation in rates of expansion and the role of conspecifics in determining patterns of settlement, including the distribution and density of nests.
4. Spatial expansion in most years was minimal, relying heavily on long‐distance dispersal of rare pioneering individuals beyond the breeding range to drive substantial recovery and thus exhibiting a stratified diffusion pattern. The distribution of nests was consequently non‐random, with strong clustering into distinct subpopulations.
5. Our findings suggest that recolonisations follow similar patterns of expansion to biological invasions, highlighting opportunities to combine knowledge across two key areas of ecological research. We also emphasise the disproportionate role of male Ospreys in restricting patterns and rates of breeding range expansion under female‐biased dispersal. Adapting conservation management accordingly could accelerate species restoration, including wider application of interventions like artificial nest provisioning and translocations to facilitate long‐distance dispersal.

## Graphical abstract

We used a unique long‐term dataset of population recovery in the Osprey *Pandion haliaetus* to reveal the similarity in spatial patterns underpinning recolonisations and biological invasions. Our findings highlight the importance of rare pioneering movements in driving range expansion, and the value of conservation interventions such as species translocations in accelerating this process.

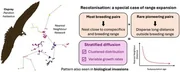

## 1 INTRODUCTION

Range expansions have many eco‐evolutionary consequences which perpetuate from the individual to the ecosystem level (Excoffier et al., 2009; Miller et al., 2020). Spatiotemporal patterns of expansion are driven by a complex suite of contextual, genetic and phenotypic mechanisms (Hengeveld, 1988). In particular, the dispersal kernel (i.e. frequency of different dispersal strategies) plays a key role in determining spatial patterns at the range front (Benton & Bowler, 2012). Shigesada and Kawasaki (2002) identified distinct range expansion patterns resulting from dispersal kernel variation and described them mathematically using stratified diffusion models. While their theoretical basis has been extensively tested in experimental settings (e.g. Goldstein et al., 2019), real‐world examples remain limited, and their proximate drivers poorly understood. Studies on mechanisms and consequences have focused on three core topics: biological invasions (Fisher, 1953; Johnson et al., 2006), spread of diseases (Murray et al., 1986) and climate‐driven expansions (Thomas & Lennon, 1999). Meanwhile recolonisations, an increasing conservation priority globally (Seddon et al., 2007), remain widely overlooked.

Commonly the focal species for recolonisations are large, long‐lived birds or mammals which were persecuted to extinction in parts of their historical breeding range (Ripple et al., 2013). Many regulate food webs, provide ecosystem services and deliver socioeconomic benefits (Sergio et al., 2008; Thompson et al., 2021). Recognition of this value, alongside modern protections and changing attitudes, has created opportunities for many species to return to their historical ranges (Lubina & Levin, 1988). In some cases, interventions such as translocations or provision of artificial breeding sites are used to aid recovery, requiring considerable expense and effort (Dennis, 2008; O'Rourke, 2014). However, the efficacy of such interventions and overall conservation of these species is limited by a lack of understanding of the mechanisms driving recovery and thus an inability to accurately predict how populations will recolonise. Licensing decisions for translocations are often based on such predictions, including anticipated rates of natural recolonisation (Trouwborst et al., 2022). If modelling fails to accurately represent the patterns underpinning range expansion, recovery rates may be overestimated, leading to missed opportunities to accelerate species restoration.

Unlike climate‐driven expansions, environmental conditions during recolonisations are more static over time, meaning habitat patch suitability may not decline with distance from the range front. The spatial patterns produced by recolonisations should therefore mirror those of biological invasions (Lensink, 1997), where the rate of expansion is determined primarily by the frequency of rare long‐distance dispersal events beyond the range front (Benton & Bowler, 2012). As such, studying patterns exhibited during recolonisations could provide greater understanding of, and ability to predict, biological invasions and vice versa.

Factors influencing settlement decisions are many and varied, and the expression and detection of individual preferences can be confounded by external influences such as patch availability and accessibility (Hanski, 1994; Nevoux et al., 2013). During recolonisations, patch availability is high and does not restrict settlement, offering a rare opportunity to investigate other factors governing individual settlement preferences. Avian recolonisations represent excellent study systems, as flying individuals can reach distant patches so are not restricted by patch accessibility. However, even large avian species often expand slowly (Lensink, 1997), indicating that additional strong factors can influence settlement decisions. These may include natal philopatry (Negro et al., 1997), conspecific attraction (Forero et al., 2002) and habitat imprinting (Stamps & Davis, 2006), although their expression varies considerably across taxa. Understanding their role in determining the settlement patterns of individual species is therefore vital for effective, targeted conservation and can contribute to wider understanding of the mechanisms driving range expansions. These concepts are poorly understood for long‐lived species, as long‐term datasets are required to conduct multigenerational studies. Here, we investigate patterns of recolonisation in a long‐lived raptor, the Osprey *Pandion haliaetus*, which has a typical lifespan of 10 years and a maximum of 27 years, with breeding from three (Mackrill, 2024).

Historically widespread across Northern Scotland (and the UK), Ospreys were locally extinct by the early 20th century, with the last confirmed breeding pair recorded in 1916 at Loch Loyne (Brown, 1979). Records suggest occasional presence thereafter, but none were confirmed breeding until 1954 when a single nest was recorded at Loch Garten, marking the start of their recolonisation and annual documentation (Dennis, 2008). The population has since recovered strongly, partly due to a concerted conservation effort involving intensive monitoring.

Here, we study the recolonisation of Northern Scotland by Ospreys during 1954–2007. Dennis (1995) observed that nests in this population appeared to group into loose colonies, as occurs across much of their global range (Poole, 1989). However, groupings were not statistically confirmed through spatial analysis and the mechanisms behind their formation were not identified. We therefore test the hypothesis that the distribution of the Northern Scotland Osprey population is not random and that nests are grouped into colonies (henceforth subpopulations). We investigate (i) temporal variation in the growth rate of the breeding Osprey population, (ii) underlying patterns of expansion and settlement and (iii) the role of conspecifics in determining nest density and spacing. We additionally test whether subpopulations were formed by a process of stratified diffusion involving both short and long‐distance dispersal, by examining the expansion rate and distribution of nests, focusing on the breeding range front.

## 2 MATERIALS AND METHODS

### 2.1 Osprey breeding behaviour

Ospreys are migratory across much of their European range, returning to breeding grounds in March and departing in September, providing a clearly defined transition into the breeding season and a considerable window for nests to be found by observers (Mackrill, 2024). Nests are often easily spotted, typically built atop prominent trees or artificial structures. Ospreys are piscivorous, diurnal hunters, commuting to water bodies several times per day, often betraying their nest location (Poole, 1989). Breeding birds also perform display flights and vocalise over their nest during courtship or if disturbed. Their nesting activities are therefore easily detected and monitored.

### 2.2 Data collection

Our dataset was collected during conservation efforts to monitor and protect recovering Ospreys across Scotland following recolonisation in 1954. From the first nest at Loch Garten (57°14′N, 3°42′W), hereafter the ‘population source’, the population expanded in all directions. We focus on the counties of Highland and Moray, covering approximately 24,000 km^2^ and capturing the fastest breeding range expansion fronts of the Northern Scotland population. Data were collected annually within this study area during 1954–2007. Beyond this period the spatial extent of the population and number of nests became too large to enable consistent monitoring, and hence, confidence in the detection of new nests and the accuracy of annual occupancy rates became too low for further years to be included in our analysis.

Osprey‐established nest sites were found through non‐systematic monitoring by conservation staff, aided by reports from landowners, land managers, volunteers and conservationists. Due to the Ospreys' size, site fidelity, conspicuous nesting activities and the high public profile of their conservation throughout the study period, we have high confidence that all nests present were detected and reported early in their occupancy. Where a new nest of unconfirmed age was found, we were able to accurately backdate occupancy from information provided by local observers in most cases. It is possible that some short‐lived nests were not discovered, but these are more likely to have been unsuitable or unsuccessful and therefore less relevant to our research questions, and so their absence is unlikely to greatly influence our findings. However, we caution that we cannot rule out bias arising from sampling effort, and some estimates of occupancy, growth and expansion may therefore be conservative. As part of conservation efforts to encourage nesting, in addition to replacing fallen and vulnerable nests at *Osprey‐established* sites, artificial nests were also erected at *human‐established* sites, generally within or close to the existing breeding range. For all nests six‐figure grid references were derived from 1:25,000 scale Ordnance Survey maps. Locations were subsequently verified using Google Earth V5.0 (Google, 2010), before extracting latitude and longitude. Where nests had multiple iterations, having been reconstructed near their original location, for example following tree fall, the most recent coordinates were used for analysis.

Known nests were surveyed annually for breeding activity, with one to six visits per year during April–August and recorded as *occupied* if two breeding adults were present. Using the *year first occupied* for each nest, we calculated *nest age* in each study year (with *year first occupied* being zero). No ethical approval or licensing permissions were required for data collection, and permissions for access were secured from the relevant landowners prior to conducting fieldwork.

### 2.3 Data analysis

Data processing and analysis was performed in R Version 4.5.1 (R Core Team, 2025). Spatial analysis was conducted using the packages *sf* (Pebesma & Bivand, 2023), *statspat.explore* (Baddeley et al., 2015) and *maptree* (Gramacy & White, 2025). Statistical analysis was conducted using the package *stats* (R Core Team, 2025), with the functions *t.test* for Welch's two sample *t*‐test (TT), *ANOVA* for analysis of variance (ANOVA), *lm* for linear models (LM) and *nls* for non‐linear least squares regressions (NLSR), including logistic regressions (LR) and negative exponential regressions (NER). Full analysis and model structures are detailed in Figure 1.

**FIGURE 1:**
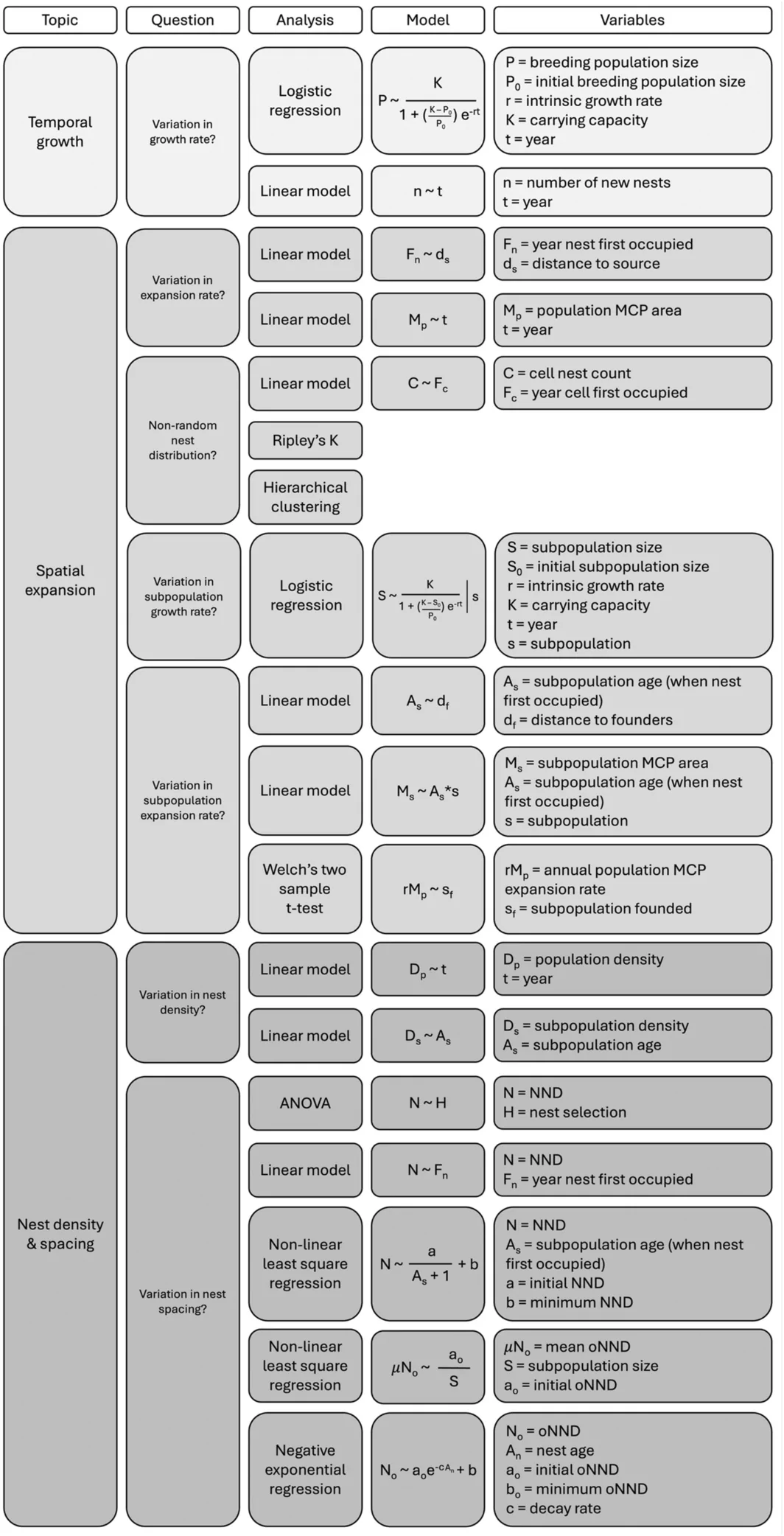
Analysis structure showing overarching topics, research questions, analysis types, models applied and variables. MCP = minimum convex polygon enclosing nests, NND = nearest neighbour distance in the year first occupied to any nest occupied in a previous year, oNND = nearest neighbour distance between nests occupied in the same year.

#### 2.3.1 Breeding population growth through time

To assess temporal growth, within each year we combined occupancy data across all nests to determine *annual breeding population size* (number of occupied nests), *cumulative breeding population size* (cumulative number of nests occupied at least once) and annual *breeding population growth rate* (annual change in occupied nests). Following Wahl and Barbraud (2014), the annual *breeding population growth factor* (r) was calculated as follows:

(1) $$r={\left(N_{t}/N_{0}\right)}^{1/t}$$

where *t* is the number of years of growth, *N*_*t*_ is the number of occupied nests at *t*, and *N*_*0*_ is the starting number of occupied nests. We modelled *annual breeding population size* against *year* using LR and extracted the predicted *carrying capacity*, *K*. We also calculated the number of *new nests occupied per year* and modelled this against *year* using LM.

#### 2.3.2 Breeding population expansion in space

Accurate spatial data was available for 96% of occupied nests. To investigate spatial expansion rates, we calculated the Euclidean *distance to the population source* from each nest using *st_distance* (*sf*) and modelled against *year first occupied* using LM. We used *st_convex_hull* (*sf*) to generate a Minimum Convex Polygon (MCP) in each year for the annual and cumulative breeding population, calculated *annual* and *cumulative MCP area* of each, respectively, using *st_area* (*sf*) and modelled this against year using LM. However, *MCP area* can be sensitive to outliers, so for more conservative estimates of breeding range size, *annual* and *cumulative MCP area* were calculated for each subpopulation and summed to give *sMCP area*. We confirmed the validity of these estimates using Kernel Density Estimation, obtaining similar results. *sMCP area* was therefore modelled against *year* using LM.

To assess spatial and temporal variations in nest distribution, a grid of 10 × 10 km cells was generated encompassing the Northern Scotland breeding population extent. *Distance to the population source*, *year first occupied* and *cumulative number of nests* per cell were calculated using *st_intersects* (*sf*). *Distance to the population source* exhibited collinearity with *year first occupied*, so *cumulative number of nests* was modelled against *year first occupied* only using LM.

We used *envelope* (*statspat.explore*) to generate a Ripley's *K* function to test for non‐random spatial distribution of nests across distances from 0 to 120 km using the default number of increments (512). The model was simulated 100 times to obtain a theoretical *K* distribution including confidence intervals. At scales where observed values lay within the predicted confidence intervals, nests were considered randomly dispersed, but where observed values deviated above or below the predicted confidence intervals, nests were considered significantly clustered or overdispersed, respectively. We generated a distance matrix for all nests using *dist* (*stats*), before using *hclust* (*stats*) to generate a hierarchical cluster tree. To obtain the optimal number of clusters, a Kelley‐Gardner‐Sutcliffe penalty function was generated using *kgs* (*maptree*), which penalises a higher number of clusters and higher mean dissimilarity within and across clusters. The hierarchical cluster tree was pruned to the size which generated the minimum penalty function value using *cutree* (*stats*). For each of the resultant groups, hereafter *subpopulation*, the *subpopulation founders* (i.e. earliest occupied nest or nests) were identified. *Year first occupied* was extracted from the *subpopulation founders*, and *subpopulation age* was then calculated for each subsequent study year.

To assess rates of subpopulation expansion we calculated *annual* and *cumulative subpopulation size*, *carrying capacity* (for the four oldest subpopulations), and *annual* and *cumulative MCP area* of each subpopulation as for the full population. Cumulative MCP of each subpopulation in the final study year was taken as their extent, and *centroids* were calculated using *st_centroid* (*sf*). We calculated the distance between the *centroids* and *edges* of all subpopulations using *st_distance* (*sf*) and extracted the *closest neighbouring subpopulation* for each. Subpopulations were considered merged if their minimum edge distance was <9.3 km: three times the mean nearest neighbour distance (NND) reported in Siverio et al. (2014). We calculated the *distance to the subpopulation founders* (in subpopulations with multiple founding nests, the first discovered was used) from all other subpopulation nests using *st_distance* (*sf*) and extracted the maximum value for each subpopulation. To test for spatial spread, *distance to the subpopulation founders* was modelled against *subpopulation age* in the year a nest was first occupied using LM. *Subpopulation MCP* area was also modelled against *subpopulation age* and *subpopulation* using LM. To assess the importance of long‐distance dispersal for spatial expansion, the population‐level *cumulative MPC expansion rate* in each year was calculated and the means of years with and without *subpopulation founders* were compared using TT.

#### 2.3.3 Nest density and spacing

To test for spatiotemporal variation in nest density, we calculated *cumulative* and *annual nest density* at both the population and subpopulation level by dividing *cumulative* and *annual MCP area* by the *cumulative breeding population size*. We modelled *cumulative* and *annual nest density* against *year* and against *subpopulation age* and *subpopulation*, respectively, using LM.

To test for spatiotemporal variation in nest spacing, we used *st_distance* (*sf*) to calculate the nearest neighbour distance (*NND*) of a nest in its *year first occupied* to any nest occupied in a previous year (i.e. the minimum distance which individuals could have dispersed to nest there). The same approach was used to calculate NND between nests occupied in the same year (*oNND*). To test for impacts of interventions, we compared *NND* of *Osprey‐* and *human‐established* nest sites using ANOVA. *NND* was modelled against *year first occupied* using LM, while at the subpopulation‐level *NND* in the *year first occupied* was modelled against *subpopulation age* and *subpopulation* using NLSR. To assess how nest spacing changed with subpopulation size we modelled *mean annual oNND* against *annual subpopulation size* using NLSR, and to investigate how nest spacing varied over the lifetime of a nest, we modelled *oNND* against *nest age* using NER.

## 3 RESULTS

### 3.1 Breeding population growth through time

During 1954–2007, 149 nests were identified, with 132 (89%) *occupied* by a breeding pair in one or more years (Figure 2), including 25 *human‐established* sites. All years except 1957 (98%) had occupied nests, with a maximum of 73 in 2004.

**FIGURE 2:**
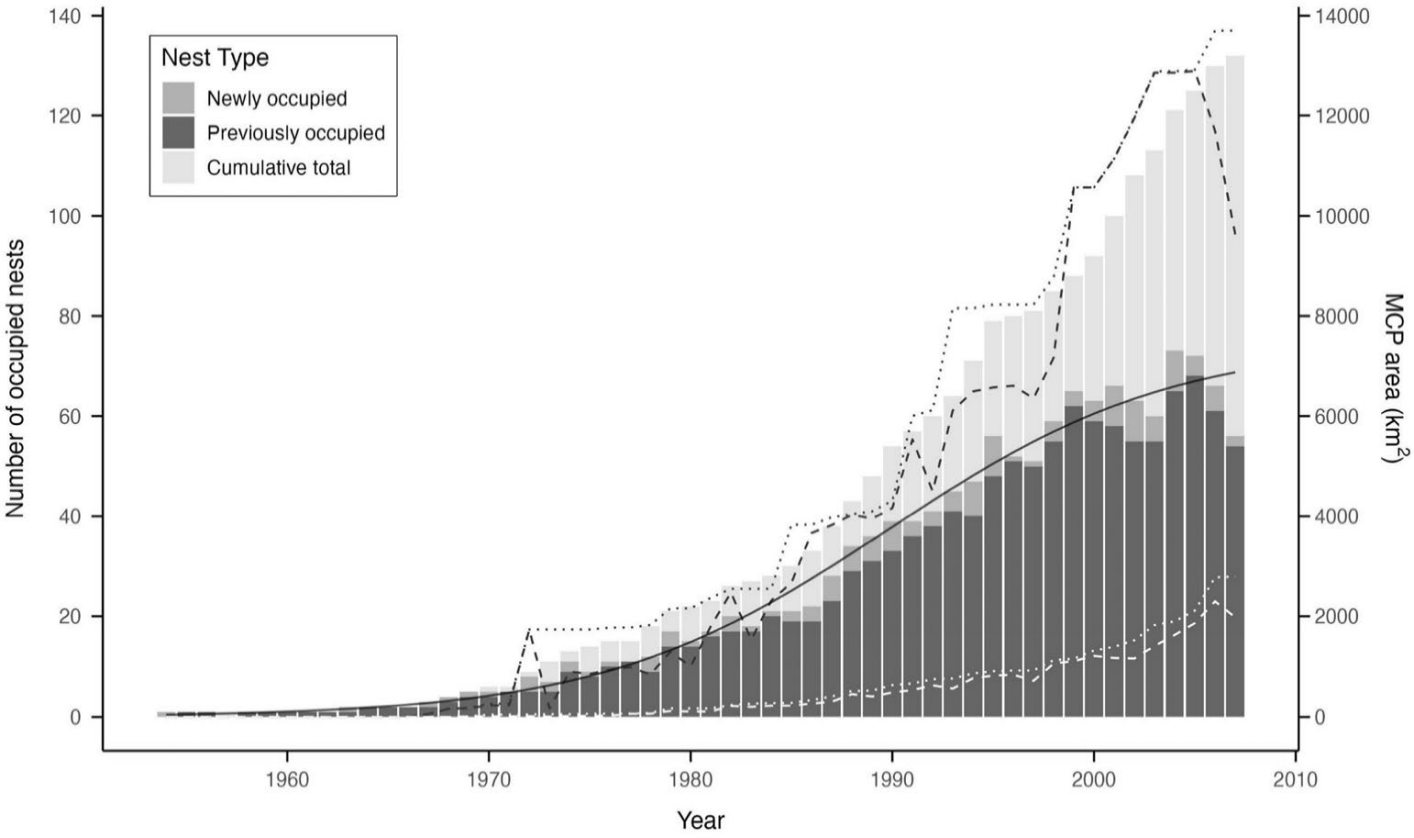
Temporal trends in breeding population size and spatial extent (1954–2007). Bars show number of nests occupied: Dark shading = nests previously occupied, medium shading = nests occupied for the first time that year. Dark and medium bars stack to give total occupied nests per year. Light shading = cumulative annual total of all nests occupied at least once, which when subtracted from the total occupied nests gives unoccupied nests per year. Solid grey line shows LR of occupied nests over time. Dashed grey line shows annual Minimum Convex Polygon (MCP) area of all occupied nests. Dotted grey line shows cumulative annual MCP area of all nests in the population occupied at least once. Dashed and dotted white lines show more conservative estimates, annual and cumulative sMCP area, respectively, calculated by summing MCP area across subpopulations.

The mean annual *breeding population growth factor* was 1.07 (SD = 0.17), and the mean annual *breeding population growth rate* was 1.0 occupied nests year^−1^ (SD = 3.6, max = 13). However, growth was not constant, exhibiting a logistic pattern (LR, *K* = 74.7, *P*_0_ = 0.46, *r* = 0.14, pseudo‐*R* ^2^ = 0.98, df = 51) with a maximum slope of 2.65 occupied nests in 1990 (Figure 2). Estimated *carrying capacity* of the breeding population was 74.7 nests (SE = 2.76) indicating that the population effectively reached carrying capacity in the final 5 years of the study. New nests were occupied in 76% of years, at a mean of 2.4 nests year^−1^ (SD = 2.4). *New nests occupied per year* increased significantly over time (LM, *β* = 0.125 ± 0.014, *F*_1,52_ = 48.08, *p* < 0.001, *R* ^2^ = 0.56), with a maximum of 8 nests year^−1^ in 1995, 2001, 2002 and 2004. Previously occupied nests became unoccupied at a mean rate of 3.1 nests year^−1^, but were reoccupied at a mean of 1.7 nests year^−1^. The *cumulative breeding population size* increased proportionately to the *annual breeding population size*, until the population approached *carrying capacity*, after which it increased linearly (Figure 2). In the final year, 58% of all previously occupied nests were unoccupied.

### 3.2 Breeding population expansion in space

During 1954–2007 the population spread to a maximum *distance to the population source* of 143 km, at a mean rate of 2.7 km year^−1^. Across all nests, *year first occupied* increased significantly with *distance to the population source* (LM, *β* = 0.17 ± 0.035, *F*_1,125_ = 25.11, *p* < 0.001, *R* ^2^ = 0.16) reflecting expansion over the study period. *Cumulative MCP area* in the final study year (i.e. the full breeding population extent) was 13,703 km^2^ (Figure 2). This increased significantly with *year* (LM, *β* = 266.82 ± 14.49, *F*_1,52_ = 339, *p* < 0.001, *R* ^2^ = 0.86), with a mean annual increase of 259 km^2^ year^−1^ (SD = 510) and a maximum of 2037 km^2^ in 1991. Estimates of breeding range area were much more conservative when calculated by summing area across subpopulations. *Cumulative sMCP area* in the final study year was 2790 km^2^ (Figure 2); increase with *year* was still significant (LM, *β* = 38.80 ± 3.31, *F*_1,52_ = 137, *p* < 0.001, *R* ^2^ = 0.72), with a mean annual increase of 53 km^2^ year^−1^ (SD = 106) and a maximum of 659 km^2^ year^−1^ in 2006. However, *cumulative MCP area* and *cumulative sMCP area* did not increase in 27 and 20 of the 54 study years, respectively (50% and 37%), indicating non‐linear expansion at both scales.

Occupied nests were not uniformly distributed at the population scale. *Cumulative number of nests* per 10 × 10 km cell across the survey area ranged from 0 to 12 and was strongly right‐skewed (median = 0; Figure 3a). *Cumulative number of nests* decreased significantly with *year first occupied* (LM, *β* = −0.1461 ± 0.0457, *F*_1,32_ = 10.22, *p* < 0.01, *R* ^2^ = 0.22), indicating that areas occupied earlier, typically closer to the population source, had more nests (Figure 3b). However, much variation remained unexplained, indicating other factors also drove distribution patterns.

**FIGURE 3:**
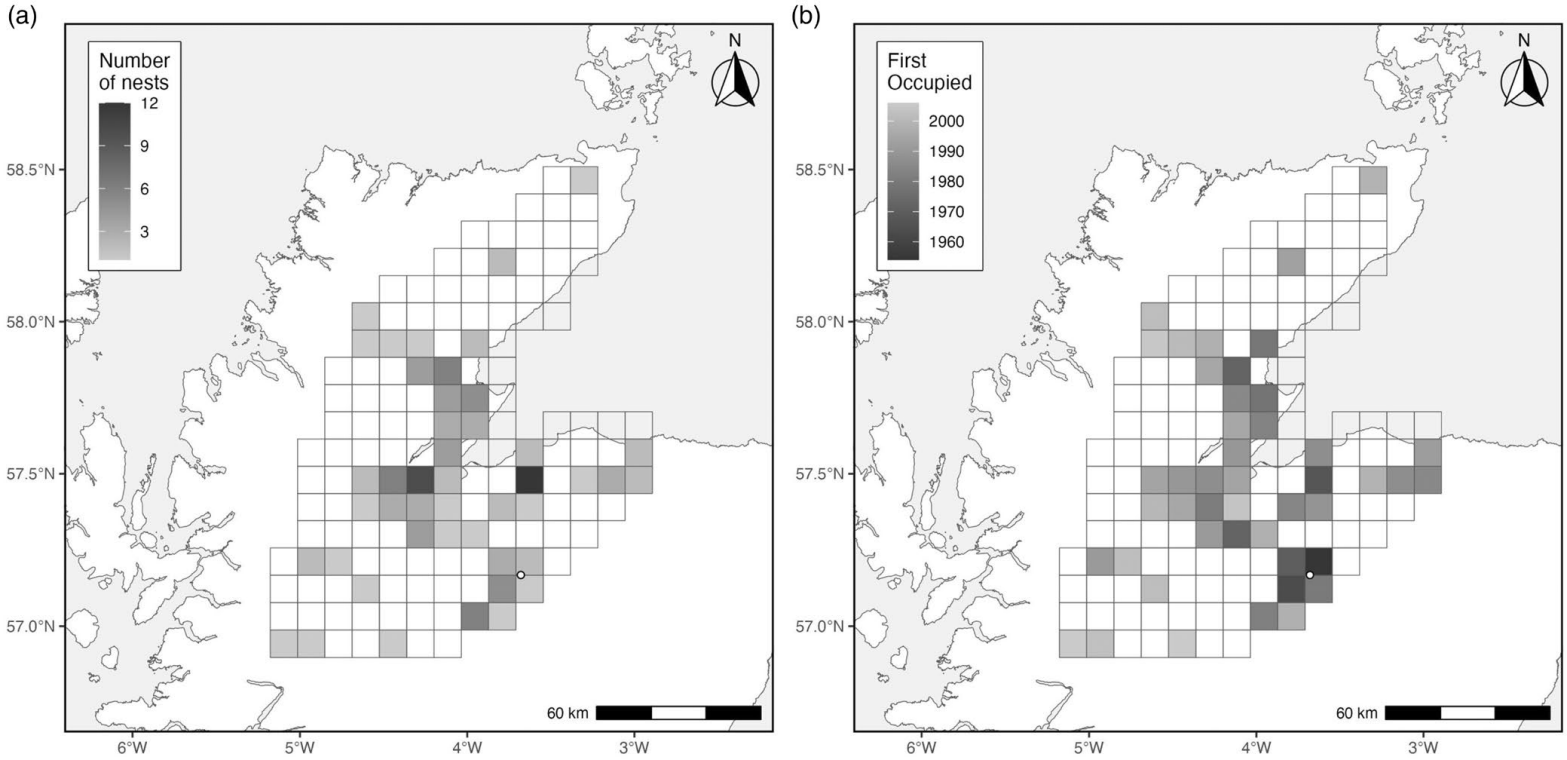
10 km grid of study area. Shading indicates (A) cumulative number of Osprey nests occupied within each square throughout the study and (B) year first occupied by Ospreys. White dot = population source.

Nests exhibited significant clustering at all distances between 703 m and 95.9 km, where Ripley's *K* deviated above the theoretical confidence intervals (Figure S1). At lower distances, nests exhibited random dispersion, while at higher distances they were overdispersed. The optimal number of subpopulations was eight (Figure 4).

**FIGURE 4:**
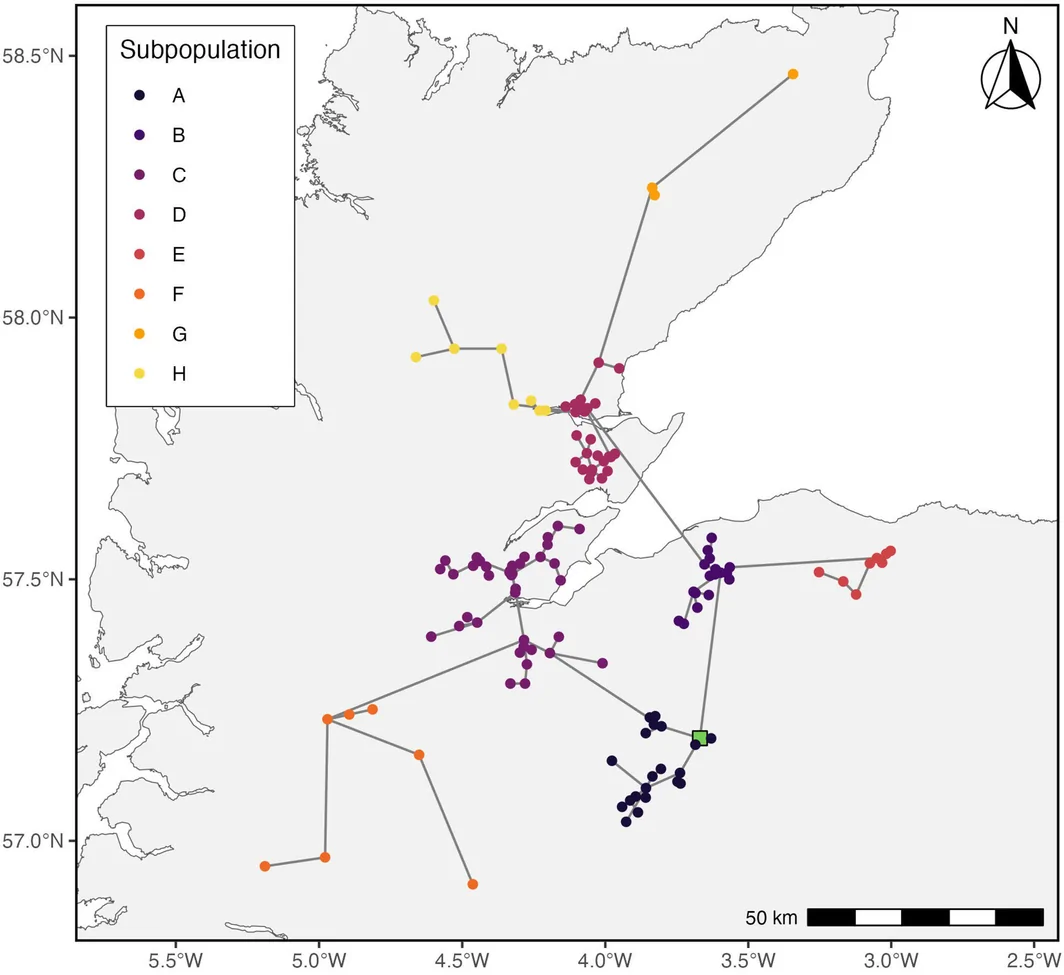
All nests (points) in study area. Black lines denote nearest neighbour relationships to previously occupied nests in the year first occupied. Point colour = subpopulation membership; green square = population source.

*Subpopulation founders* used *human‐established* nest sites in three cases (C, G & H). Subpopulations were generally founded by a single pair, except in subpopulation H when two nests were occupied simultaneously. Excluding this, the mean time for a second nest to be occupied was 7.6 years. Subpopulations contained between 3 and 38 nests that were occupied at least once (mean = 16, SD = 12) by 2007. Older subpopulations contained more nests, and a logistic growth pattern with increasing *subpopulation age*, similar to the population level (LR, Pseudo *R* ^2^ = 0.96, df = 155; Table S1, Figure 5). Mean predicted *carrying capacity* for the four oldest subpopulations was 13.6 nests. All four reached carrying capacity during the study, in a mean time of 30.5 years. *Cumulative subpopulation size* continued increasing after reaching *carrying capacity*, although more gradually than at the population level.

**FIGURE 5:**
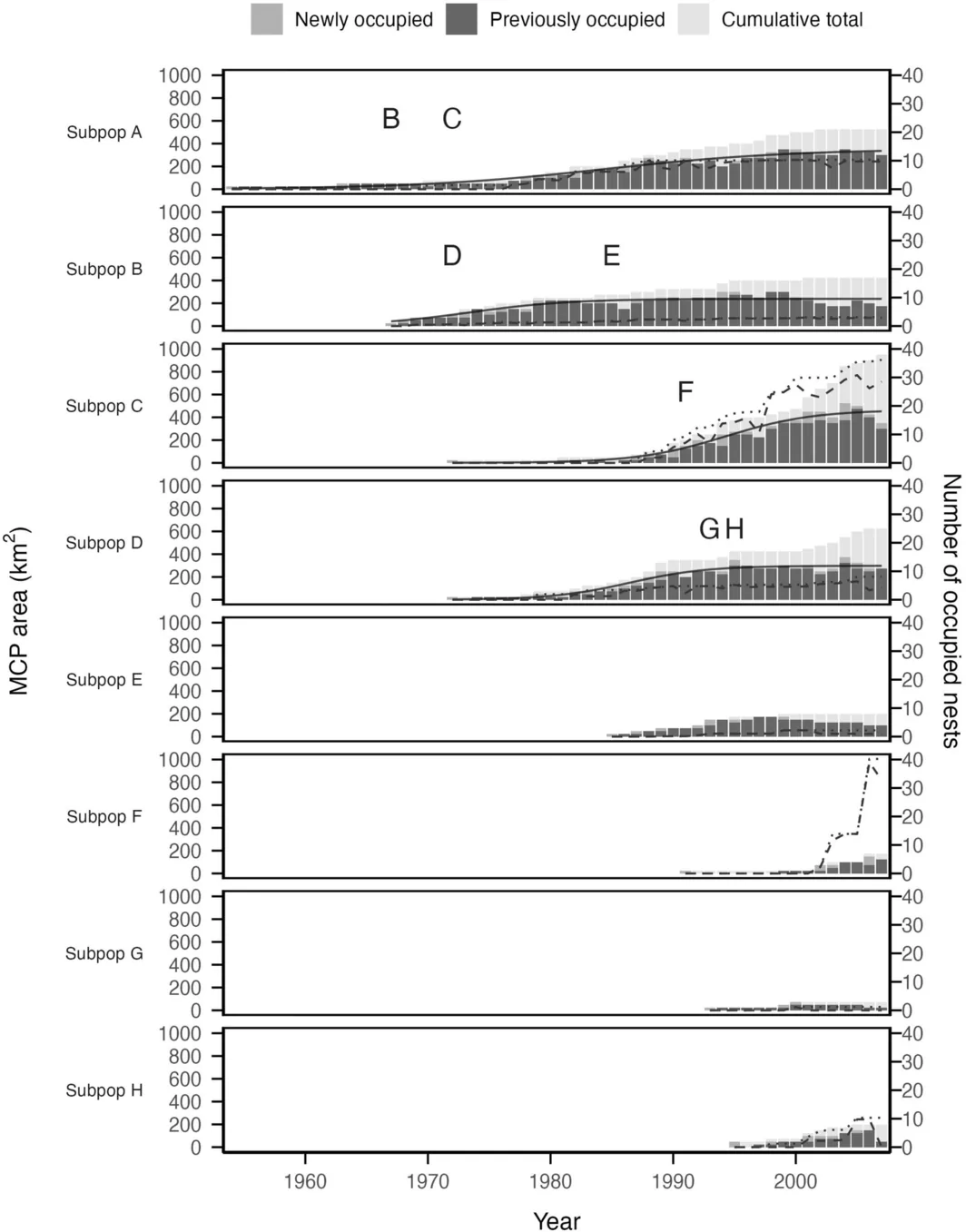
Temporal trend in subpopulation size and area (1954–2007). Bars show number of occupied nests: Dark shading = previously occupied nests, medium shading = nests occupied for the first time that year. Dark and medium bars stack to give total occupied nests per year. Light shading = cumulative annual total of all nests occupied at least once. Solid line shows LR of occupied nests over time for the four oldest subpopulations. Dashed line shows annual Minimum Convex Polygon (MCP) area of all occupied nests (km^2^). Dotted line shows cumulative annual MCP area of all nests occupied at least once (km^2^). Rows ordered by subpopulation age.

New subpopulations were founded on average every 6.75 years, but not at a constant rate. New subpopulations were established a mean of 16 years after the founding of their *closest neighbouring subpopulation* (the most likely subpopulation of origin for *subpopulation founders*), and before the *closest neighbouring subpopulation* had reached *carrying capacity* (Figure 5). All eight subpopulations contained occupied nests in the final study year, indicating no permanent subpopulation extinction. However, temporary unoccupancy was observed in subpopulations, typically in the first decade and no later than 13 years after founding. The mean number of years unoccupied once founded was 2.5 years; however, in two subpopulations (C and F), the *year first occupied* was immediately followed by 10 and 7 years, respectively, of no occupancy before the founding nest was reoccupied or another was established.

Mean distance between the *edges* and *centroids* of *closest neighbouring subpopulations* was 16.1 km (SD = 10.6, *n* = 8) and 39.8 km (SD = 12.1, *n* = 8), respectively. Distance between *edges* of *closest neighbouring subpopulations* decreased over time, however, only subpopulations D and H merged (minimum edge distance = 4.4 km). *Distance from the subpopulation founders* increased significantly with *subpopulation age* (LM, *β* = 163.83 ± 72.68, *F*_1,116_ = 5.082, *p* = 0.026, *R* ^2^ = 0.03); however, the model failed to explain much of the variation, including continued occupation of new nests infilling close to the founding nest after several decades (Figure S2). Maximum *distance to the subpopulation founders* was 46.9 km. *Subpopulation MCP area* also increased significantly with *subpopulation age* (LM, *F*_1,219_ = 97.2, *p* < 0.001, *R* ^2^ = 0.11, Table S2), but mean area (*F*_7,219_ = 10.8, *p* < 0.001, *R* ^2^ = 0.08) and the effect of age (*F*_7,219_ = 10.8, *p* < 0.001, *R* ^2^ = 0.53) varied significantly by *subpopulation*, increasing at a faster rate in subpopulations C, F and H and slower in subpopulation B (Table S3; Figure 5). *Cumulative MCP area* in 2007 of subpopulations that reached *carrying capacity* ranged from 77.6–904 km^2^ (Figure 5), all having continued to increase after reaching *carrying capacity*, although some only marginally. Mean increase in subpopulation *annual MCP area* was 12.3 km^2^ year^−1^ (SD = 52.1); however, the mean percentage of years with no expansion per subpopulation was higher than at the population level at 75% (SD = 12.2). Permanent contractions in subpopulation breeding range were rare, although temporary contractions were observed where sites became unoccupied. Population‐level *cumulative MPC expansion rate* was significantly higher in years with *subpopulation founders* (mean = 1102 km^2^ year^−1^) than those when they were not (mean = 151 km^2^ year^−1^; TT, *t* = −2.74, df = 5.21, *p*‐value = 0.039).

### 3.3 Nest density and spacing

At the population level, *cumulative nest density* increased over time (LM, *β* = 0.005 ± 0.002, *F*_1,34_ = 7.07 *p* = 0.012, *R* ^2^ = 0.15), from 0.51 nests per 100 km^2^ in 1972 to 0.92 nests per 100 km^2^ in 2007. By contrast, *annual nest density* declined over time (LM, *β* = −0.018 ± 0.003, *F*_1,32_ = 42.63, *p* < 0.001, *R* ^2^ = 0.56), from 1.0 nest per 100 km^2^ in 1984 to 0.58 nest per 100 km^2^ in 2007. At the subpopulation level, mean *cumulative nest density* was 12.4 nests per 100 km^2^ (SD = 8.6), while mean *annual nest density* was 11.2 nests per 100 km^2^ (SD = 8.2). *Annual nest density* did not vary significantly with *subpopulation age* (LM, *F*_1,151_ = 0.01, *p* = 0.923, *R* ^2^ = 0.00; Table S4), but mean *annual nest density* (*F*_7,151_ = 94.6, *p* < 0.001, *R* ^2^ = 0.63) and the effect of *subpopulation age* (*F*_7,151_ = 27.4, *p* < 0.001, *R* ^2^ = 0.18) varied by *subpopulation*, with higher density in subpopulations B and E but declining towards the average over time (Table S5).

Median *NND* was 3.0 km (*n* = 126) with a long‐tailed leptokurtic distribution, ranging from 315 m to 45.2 km (Figure S3). Median *NND* was lower for *Osprey‐established* (2.8 km) than *human‐established* nest sites (4.4 km), but not significantly so (ANOVA, *F*_1,124_ = 1.179, *p* = 0.28; Figure 6). At the population level, *NND* showed no trend with *year* (LM, *β* = −132.30 ± 76.89, *F*_1,123_ = 15.84, *p* = 0.088, *R* ^2^ = 0.19), but at the subpopulation‐level *NND* exhibited a negative hyperbolic relationship with *subpopulation age* (NLSR, Pseudo‐*R* ^2^ = 0.79, df = 110; Table S6, Figure 7). *Subpopulation founders* had the highest *NND* of any nest in all subpopulations (mean = 33.8 km, SD = 10.9 km), and the *NND* of all other nests (mean = 4.7 km, SD = 5.8 km) rapidly declined with *subpopulation age* towards a mean minimum threshold of 1.4 km. This asymptotic trend was consistent across subpopulations, except for the three most recently founded subpopulations: H was established close to an existing subpopulation (D) and nests therefore had low *NND* regardless of *subpopulation age*, and subpopulations F and G exhibited weaker clustering than the other subpopulations.

**FIGURE 6:**
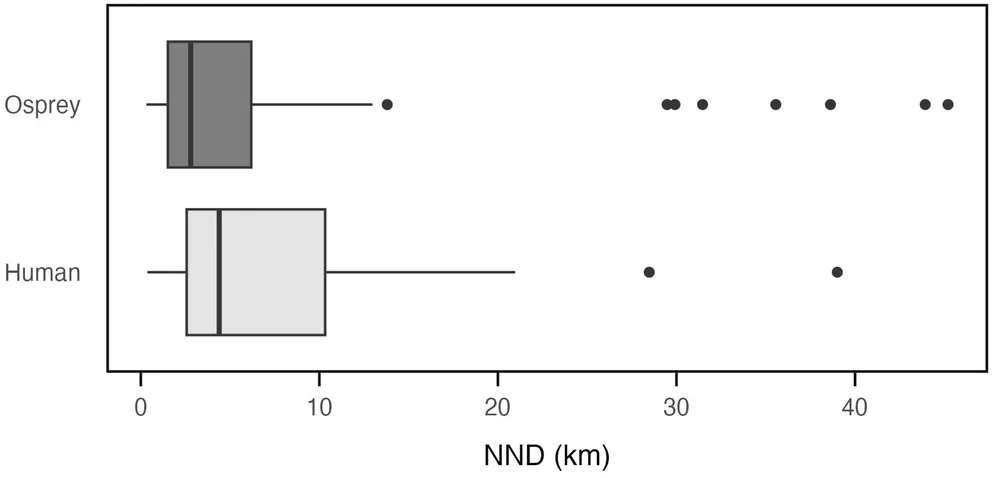
Nearest neighbour distance (NND) to previously occupied nests in year first occupied for Osprey‐ and human‐established nest sites.

**FIGURE 7:**
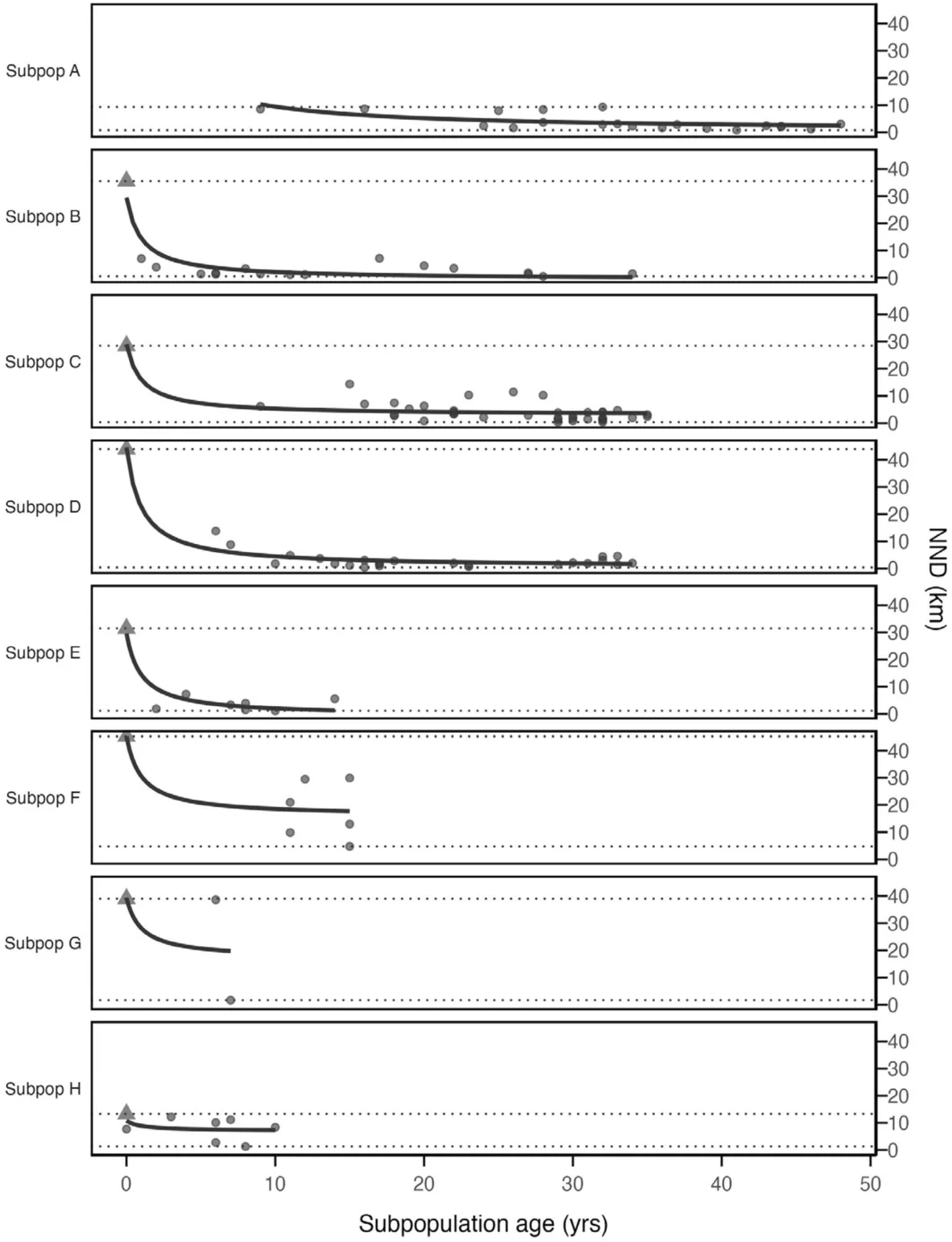
Nearest neighbour distance (NND) to previously occupied nests in year first occupied against subpopulation age. Triangles = subpopulation founders (omitted for subpopulation A as NND unknown), points = subsequent nests. Solid lines show NLSRs fitted to data for each subpopulation separately. Dotted lines show maximum and minimum NNDs in each subpopulation.

*oNND* ranged from 316 m to 43.9 km, with a median of 2.18 km. *Mean annual oNND* exhibited a negative hyperbolic relationship with *annual subpopulation size* (NLSR, *a*_o_ = 34.0 km, Pseudo *R* ^2^ = 0.76, df = 206; Figure S4). At the individual nest level, *oNND* also declined with *nest age* towards a threshold of ~2.75 km; however, variance was high and only a small proportion was explained by the model (NER, *a*_o_ = 4.75 km, *b*_o_ = 0.11 km, *c* = 2.75, Pseudo *R* ^2^ = 0.04, df = 1263; Figure S5).

## 4 DISCUSSION

### 4.1 Demographic patterns

The patterns underpinning recolonisations are complex and have significant consequences for expansion rates in a recovering species. The Northern Scotland Osprey population exhibited strong recovery over half a century. Logistic growth at the breeding population level suggests a carrying capacity was reached within the study area; however, occupation of new nests continued at a constant rate thereafter, highlighting ongoing nest turnover. As breeding dispersal is rare in Ospreys, this is likely driven by (1) desertion of failed nest sites, particularly by inexperienced pairs and/or (2) turnover of breeding individuals (Mackrill, 2024). Young birds often prefer to take over existing nests, which likely explains the high uptake of human‐established sites (19% of occupied nests). However, new nests may be established by new pairs through choice or by necessity, or by breeding individuals who lose their original mate and re‐pair (Poole, 1989). Deteriorating local conditions could also lead to nest turnover, although this is unlikely to be a key driver here, as most sites became reoccupied later. Logistic growth was also observed at the subpopulation level, with older subpopulations reaching a local carrying capacity. This was generally consistent across subpopulations, suggesting an optimal subpopulation size in Northern Scotland which maximises the trade‐off between conspecific attraction and resource availability (Lõhmus, 2001).

### 4.2 Range expansion

Spatial recovery was also substantial, and we report the first accurate expansion rates for breeding Ospreys, providing a baseline for evaluation elsewhere. The population‐level mean was high (259 km^2^ year^−1^), but when calculated from subpopulation‐level data was considerably lower (53 km^2^ year^−1^) and likely more representative of the true area recovered per year. At the subpopulation level, expansion rates were constant over time but varied between subpopulations, suggesting spatial variation in the drivers of expansion. However, given the Osprey's capacity for long‐distance movements (Poole, 1989) and the substantial size of our population, the expansion rate of Ospreys may overall be considered slow (Lõhmus, 2001). In conservation terms, our findings indicate that natural range recovery on national or international scales could take decades, highlighting the importance of conservation interventions which accelerate expansion.

Overall breeding range expansion was non‐linear, characterised by periods of minimal change punctuated by occasional large increases. Rather than reflecting short‐distance dispersal beyond the range front in multiple places, the years with significant expansion (and thus the majority of spatial recovery) were driven almost entirely by long‐distance dispersal of a small number of pioneering pairs outside the existing range. Subsequent expansion centred around these pioneers, creating a stratified diffusion pattern (Shigesada & Kawasaki, 2002), strongly supporting our hypothesis that breeding range expansion during recolonisation mirrors that of biological invasions (Bowler & Benton, 2005).

### 4.3 Pioneering dispersal

The scarcity of pioneering movements may be explained by two key individual‐level drivers. Conspecific attraction is exhibited by both sexes and drives settlement close to existing breeding pairs (Lõhmus, 2001). In males, this preference is often masked by strong natal philopatry, resulting in shorter dispersal distances (Poole, 1989). Since males are often responsible for selecting a nest site, to which they attract a mate (Dennis, 2008), we suggest that the frequency of pioneering *males* in particular limits the breeding range expansion rate. This has broad implications for Osprey population management, suggesting we should focus on encouraging male dispersal beyond the breeding range. Three founding pairs used human‐established sites, indicating that artificial nest provisioning can help facilitate pioneering expansion, although data was insufficient to determine whether it accelerated the process. In England, artificial nests alone have been sufficient to drive recolonisation of some counties, but in others translocation has been necessary (Mackrill, 2024). Sex ratios of released birds are often biased towards males, but our findings suggest that this should be increased further. Initial population growth may still be limited by female availability, but translocating more females does little to remedy this due to low philopatry rates (Mackrill et al., 2013). Conspecific attraction is likely of greater importance to increasing female availability, and therefore, proximity to sources of immigrants such as neighbouring populations and migratory flyways should increase expansion rates.

Long‐distance dispersal may be rare in male Ospreys for a variety of reasons. High female dispersal propensities may adequately reduce inbreeding risks such that non‐dispersing males avoid associated fitness consequences (Greenwood et al., 1978). Long‐distance dispersal also carries risks, including energetic and time costs of searching for nest sites or mates and opportunity costs of losing familiarity or competitive advantages (Brown et al., 2008). However, while familiarity and likelihood of finding a mate may be higher closer to the existing breeding range, unpaired sub‐adult Ospreys are highly nomadic, first returning to northerly latitudes in their third year and ranging over hundreds of kilometres (Mackrill, 2024). This provides substantial opportunity to search for potential nest sites and mates, and acquire a more complete knowledge of patch quality, including the distribution and competitive ability of conspecifics. Settlement is therefore likely an informed decision, and the adaptive value of limited male dispersal remains unclear. It may instead be a neutral or negative artefact of other mechanisms, for example imprinting, natal habitat recognition or migratory navigation. Indeed, contradiction between settlement preferences and fitness consequences has been identified elsewhere: Bai et al. (2009) found that German Ospreys overexploited poor‐quality habitats. This warrants further investigation using individual dispersal data in future studies.

Although rare, we show that long‐distance dispersing males do exist and play a vital role in facilitating significant breeding range expansion. Identifying what drives expression of these rare phenotypes is therefore key to understanding and accelerating recolonisation. Known drivers include avoidance of competition or deteriorating local conditions (Hamilton & May, 1977; Ronce, 2007). Thus, pioneering movements may result from a ‘pushing’ influence, such as a lack of suitable nest sites or competition at high densities within the existing breeding range. However, our findings refute this hypothesis, with subpopulation expansion continuing long after new subpopulations were established nearby and with new nests appearing close to founding nests even 30 years after establishment, suggesting density had not yet peaked and pioneering birds actively chose not to occupy available suitable sites. Indeed, nests were often established in the space between subpopulation founders and the closest neighbouring subpopulation, implying that pioneering individuals passed suitable patches before settling. This may have been driven by a ‘pulling’ influence, such as attraction to higher quality patches (Fretwell & Lucas, 1970) or particular habitats (e.g. Natal Habitat Imprinting; Davis & Stamps, 2004). Greater territoriality of established breeders at lower densities may be another ‘pushing’ influence affecting the timing of pioneering dispersal. Satellite tracking of Ospreys has shown that males will chase competitors over hundreds of kilometres, well beyond the breeding range (Mackrill, 2024): energetically costly behaviour which can only be employed when competitor numbers are low. Alternatively, pioneering dispersal may result from a weak ‘pulling’ influence from the existing breeding range. This may be driven by contextual cues such as conspecific attraction, which is weaker in smaller subpopulations, explaining why subpopulations were usually established in the first two decades after their closest neighbouring subpopulation was founded. Evidence from the Osprey translocation at Rutland Water corroborates this theory, where two males released in the 1990s independently dispersed over 150 km to recolonise Wales in 2004. They may have experienced limited conspecific attraction during their juvenile and sub‐adult phases due to a lack of breeding adults at their de facto natal site, resulting in long‐distance dispersal and thus substantial range expansion (Mackrill, 2024).

### 4.4 Semi‐coloniality

We provide the first statistical evidence supporting Dennis' (1995) observation that Scottish Osprey nests appear grouped into loose colonies. This aligns with semi‐coloniality exhibited by Ospreys elsewhere, although density varies globally, with the highest being in the United States and Mexico where multiple pairs can nest in a single tree (Poole, 1989). Density was generally higher where nests had been established longer but did not vary greatly between subpopulations or with subpopulation age, suggesting optimal density may exist within Osprey populations. Much of the unexplained variation in density may also have been driven by underlying habitat availability or quality, meriting further investigation.

All subpopulations except one were founded by pioneering pairs. While NND exhibited no temporal pattern at the population level, it showed a clear negative asymptotic relationship with subpopulation age, like that observed in colonial Cliff swallows (*Petrochelidon pyrrhonota*) (Brown & Brown, 2000). The three latest established subpopulations were exceptions; however, this is possibly an artefact of the clustering method, and if re‐examined with additional years of data, some members of these subpopulations could be considered founders of their own subpopulations. Spatial clustering alone thus cannot fully capture development of subpopulation structure over time, and future analyses incorporating approaches such as weighted clustering with temporal variables may remedy this. The rapid decline in oNND with subpopulation size suggests later established pairs more frequently chose to nest within or closer to the existing subpopulation range, gradually infilling gaps over time. This was further supported by the decline in oNND with nest age, suggesting pairs chose to settle away from conspecifics but later gained closer neighbours. Such increasing density may be driven by (1) occupation of progressively lower quality sites with declining availability, (2) positive feedback of conspecific attraction in high density areas or (3) reductions in territoriality with increasing competitors, such that sites inaccessible to earlier pairs become available later.

Subpopulations were consistently occupied, suggesting that once an area is recolonised it will be used long‐term. Breeding range contractions were rare and temporary, indicating stability in Osprey metapopulation dynamics, with no substantial source‐sink relationships besides those during expansion (Hanski, 1994). Subpopulations did not generally merge, despite reaching considerable sizes and expanding linearly, possibly due to (1) insufficient time elapsing, (2) lower quality patches existing between subpopulations or (3) density‐dependent resource limitation. Indeed, spatial expansion tailed off at carrying capacity, again suggesting optimal size limits may exist, although these varied between subpopulations.

Initial subpopulation growth was slow and likely dependent on two non‐mutually exclusive mechanisms: (1) natal philopatry of the founding pair's offspring and (2) further immigration into the area. As Ospreys exhibit sex‐biased dispersal, males and females are expected to dominate the first and second strategies, respectively (Poole, 1989). Male availability would therefore be limited by generational timings (minimum breeding age is 3 years old; Dennis, 2008), resulting in slow initial growth. Fewer males may also lead to lower female immigration where conspecific attraction drives dispersal, suggesting that while female availability in the wider metapopulation may impact subpopulation growth rates, the availability of males is again the main limiting factor. However, natal philopatry in this case assumes direct contradiction between dispersal behaviour of pioneering parents and their male offspring, implying this is not a heritable trait. Were dispersal heritable, we might expect growth to be slower still, as most male offspring of pioneers would emigrate from their natal subpopulation, thus relying on immigration of further rare pioneering males and on stochastic variation in dispersal characteristics to facilitate growth. While evidence supporting this theory is limited and mixed, it cannot be dismissed in Northern Scotland, as some subpopulations took over a decade to grow, and the fastest expanding subpopulation was founded closest to another, suggesting immigration rates are a key limiting factor. These hypotheses merit further investigation employing genealogies and individual‐level dispersal data.

### 4.5 Broader implications

Our analysis reveals the complexity of settlement patterns during species recolonisation and the consequent unpredictability of range expansion. However, several key findings give new insights into underlying mechanisms, with potential benefits to the wider study of spatial population dynamics and applications for species conservation.

The scarcity of pioneering movements and non‐linearity of expansion rates has implications for accurate modelling of range expansion. Existing approaches such as dynamic Species Distribution Models (SDMs) attempt to account for such patterns by incorporating species‐specific dispersal kernels to predict spatial expansion (Holloway et al., 2016). However, our findings demonstrate that in some species patterns of dispersal at the range front are not equivalent to the wider population and individuals settle within the existing range more frequently than expected by chance, likely due to conspecific attraction. Additionally, the timing of pioneering movements can be non‐random, with long‐distance expansion most likely to occur in Scottish Ospreys one to two decades after colonisation of a neighbouring area. Dispersal distances alone are therefore insufficient to capture dynamics at the range expansion front and likely result in overestimation of recovery rates. The need for models which effectively incorporate conspecific attraction alongside other variables influencing settlement, such as habitat availability and dispersal, has long been emphasised (Campomizzi et al., 2008) but examples remain limited, highlighting a key area for future research.

The same findings also highlight the need for conservation interventions which replicate or accelerate pioneering movements to enhance spatial recovery of species exhibiting conspecific attraction. For example, translocation is an effective, widely used tool (Trouwborst et al., 2022) but its application is often rejected based on insufficient evidence, such as inadequate modelling or extrapolation of simple statistics like mean expansion rates to predict windows of natural recolonisation, which greatly overestimate range recovery speeds. We therefore encourage researchers, conservationists and policy makers to pay greater consideration to species‐specific expansion patterns when making conservation management decisions, and to recognise the value of translocation and breeding site provisioning as methods for accelerating the restoration of lost species, by applying these tools with greater frequency and urgency.

The disproportionate role of male dispersal in determining both the fundamental distribution of populations and the recovery rate in species with female‐biased dispersal has potential eco‐evolutionary implications, including individual‐ and population‐level feedbacks (Miller et al., 2020). Experimental approaches could be applied to interrogate these drivers and consequences, requiring investigation in other taxa with sex‐biased dispersal. This finding also reinforces the need for targeted conservation measures, suggesting that male dispersal is a key factor limiting range expansion for species with female‐biased dispersal and increasing the rate of male pioneering movements (e.g. via translocation) should be a management priority.

Finally, the similarity in underlying patterns of spatial expansion between recolonisations and biological invasions has implications for the understanding of both processes. Priority should be placed on investigating spatial recovery in other species to assess whether our finding is applicable across taxa and regions. The spatial dynamics of recolonisations remain poorly understood; however, biological invasions have been subject to extensive research for many decades, and thus a broad knowledgebase has been amassed on their drivers, mechanisms and patterns (Andrade‐Restrepo et al., 2019; Haubrock et al., 2025; Hengeveld, 1988), alongside modelling to predict their consequences (Mainali et al., 2015). There is therefore great potential, if similarities between the two processes prove ubiquitous, to draw on this resource to address research questions surrounding recolonisations and thus rapidly expand our understanding. In particular, application of existing models (Belouard et al., 2025; Dominguez Almela et al., 2020) to predict recolonisation probabilities and patterns has considerable potential to improve the accuracy and efficacy of conservation management for recovering species.

## AUTHOR CONTRIBUTIONS

Roy Dennis collected the data; Brittany C. Maxted conceived and designed the methods, analysed the data and led the writing of the manuscript. Richard A. Stillman, Phillipa K. Gillingham, Ross A. Hill, Tim Mackrill and Roy Dennis contributed to methods development, data interpretation and manuscript drafts and gave final approval for publication.

## CONFLICT OF INTEREST STATEMENT

The authors declare no conflict of interest.

## STATEMENT ON INCLUSION

All authors are UK‐based and the author who performed fieldwork is Scotland‐based. Local community engagement was central throughout, with many contributing to data collection and receiving acknowledgement. Publication language is the primary language spoken locally, ensuring findings are accessible to locals, interested parties and contributors.

## Supporting information


**Figure S1.** Ripley's *K* function for the population nest point pattern: *r* = distance (m), *K*(*r*) = Ripley's *K* value at a given distance, *K*_obs_ = observed *K*, *K*_theo_ = theoretical *K* under a random distribution, *K*_hi_ & *K*_lo_ = upper and lower limit of confidence interval for theoretical *K*, respectively. Deviations above and below the predicted confidence intervals indicate statistically significant clustering and dispersion, respectively.
**Figure S2**. Distance to subpopulation founders (km) with subpopulation age (years). Points show individual nests; line shows fitted linear model with (shaded) confidence intervals.
**Figure S3**. Density kernel of Nearest Neighbour Distance to previously occupied nests in the first year of occupancy.
**Figure S4**. Mean nearest neighbour distance to other occupied nests (oNND) against subpopulation size. Dots represent raw values, line represents fitted non‐linear least square model.
**Figure S5**. Nearest neighbour distance to other occupied nests (oNND) against nest age. Dots and bars represent mean ± confidence intervals at each age, line represents fitted linear model with (shaded) confidence intervals.
**Table S1**. Parameter estimates for the Logistic Regression (LR) of subpopulation size against subpopulation age, where *K* is the subpopulation carrying capacity, *S*_0_ is the initial subpopulation size (km) and *r* is the intrinsic growth rate.
**Table S2**. ANOVA summary of the Linear Model (LM) examining the overall effects of subpopulation age, subpopulation and their interaction on Minimum Convex Polygon (MCP) area. Shown are the Sum of squares (Sum Sq), degrees of freedom (Df), the test statistic *F* and the *p* values.
**Table S3**. Summary of the Linear Model (LM) examining the effect of subpopulation age and subpopulation on Minimum Convex Polygon (MCP) area. Shown are estimates, standard errors, the test statistic *t* and the *p* values.
**Table S4**. ANOVA summary of the Linear Model (LM) examining the overall effects of subpopulation age, subpopulation and their interaction on nest density. Shown are the Sum of squares (Sum Sq), degrees of freedom (Df), the test statistic *F* and the *p* values.
**Table S5**. Summary of the Linear Model (LM) examining the effect of subpopulation age and subpopulation on nest density. Shown are estimates, standard errors, the test statistic *t* and the *p* values.
**Table S6**. Parameter estimates for the Non‐linear Least Squares Regression (NLSR) of Nearest Neighbour Distance (NND) against subpopulation age, where a is the initial NND (km) and b is the minimum NND (km).

## Data Availability

Data has not been made available due to its sensitive nature and will only be available upon request under the terms of a usage agreement with the Roy Dennis Wildlife Foundation.
