# Supplementary material for: Long‐term patterns of breeding range expansion mirror invasion dynamics during recolonisation of a semi‐colonial raptor
Source: J Anim Ecol. 2026 Aug 12;95(10):2013–27. doi: 10.1111/1365-2656.70322 (PMC13643183; doi:10.1111/1365-2656.70322)
Supplement: Supplementary file 1 [file JANE-95-2013-s001.docx]

## **Supplementary Information**

*Figure S1. Ripley’s K function for the population nest point pattern: r = distance (m), K(r) = Ripley’s K value at a given distance, K_obs_ = observed K, K_theo_ = theoretical K under a random distribution, K_hi_ & K_lo_ = upper and lower limit of confidence interval for theoretical K respectively. Deviations above and below the predicted confidence intervals indicate statistically significant clustering and dispersion respectively.*

*Table S1. Parameter estimates for the Logistic Regression (LR) of subpopulation size against subpopulation age, where K is the subpopulation carrying capacity, S_0_ is the initial subpopulation size (km) and r is the intrinsic growth rate.*

| Subpopulation | K | S_0_ | r |
| --- | --- | --- | --- |
| A | 14.16 | 0.26 | 0.13 |
| B | 9.55 | 1.65 | 0.24 |
| C | 18.61 | 0.04 | 0.28 |
| D | 11.93 | 0.12 | 0.32 |

*Table S2. ANOVA summary of the Linear Model (LM) examining the overall effects of subpopulation age, subpopulation and their interaction on Minimum Convex Polygon (MCP) area. Shown are the Sum of squares (Sum Sq), degrees of freedom (Df), the test statistic F, and the p values.*

| Coefficient | Sum Sq | Df | F value | p |
| --- | --- | --- | --- | --- |
| (Intercept) | **41933.37** | **1** | **6.72** | **0.01** |
| Subpopulation age | **606308.85** | **1** | **97.17** | **< 0.001** |
| Subpopulation | **473348.23** | **7** | **10.84** | **< 0.001** |
| Subpopulation age:Subpopulation | **2848522.91** | **7** | **65.22** | **< 0.001** |
| Residuals | **1366505.77** | **219** | **NA** | **NA** |

*Table S3. Summary of the Linear Model (LM) examining the effect of subpopulation age and subpopulation on Minimum Convex Polygon (MCP) area. Shown are estimates, standard errors, the test statistic t, and the p values.*

| variable | Estimate | Std. Error | t value | p |
| --- | --- | --- | --- | --- |
| Intercept | **-54.97** | **21.2** | **-2.59** | **0.01** |
| Subpopulation age | **6.8** | **0.69** | **9.86** | **< 0.001** |
| Subpopulation B | **62.94** | **32.2** | **1.95** | **0.052** |
| Subpopulation C | **-167.51** | **33.39** | **-5.02** | **< 0.001** |
| Subpopulation D | **52.18** | **33.39** | **1.56** | **0.12** |
| Subpopulation E | **48.34** | **38.3** | **1.26** | **0.208** |
| Subpopulation F | **-163.28** | **42.37** | **-3.85** | **< 0.001** |
| Subpopulation G | **49.97** | **44.24** | **1.13** | **0.26** |
| Subpopulation H | **11.82** | **46.52** | **0.25** | **0.8** |
| Subpopulation age:Subpopulation B | **-4.83** | **1.25** | **-3.87** | **< 0.001** |
| Subpopulation age:Subpopulation C | **22.99** | **1.44** | **15.93** | **< 0.001** |
| Subpopulation age:Subpopulation D | **-1.18** | **1.44** | **-0.82** | **0.414** |
| Subpopulation age:Subpopulation E | **-3.54** | **2.58** | **-1.37** | **0.171** |
| Subpopulation age:Subpopulation F | **43.34** | **3.97** | **10.91** | **< 0.001** |
| Subpopulation age:Subpopulation G | **-3.8** | **4.77** | **-0.8** | **0.427** |
| Subpopulation age:Subpopulation H | **18.39** | **5.9** | **3.12** | **0.002** |

*Figure S2. Distance to subpopulation founders (km) with subpopulation age (years). Points show individual nests; line shows fitted linear model with (shaded) confidence intervals.*

*Table S4. ANOVA summary of the Linear Model (LM) examining the overall effects of subpopulation age, subpopulation and their interaction on nest density. Shown are the Sum of squares (Sum Sq), degrees of freedom (Df), the test statistic F, and the p values.*

| Coefficient | Sum Sq | Df | F value | p |
| --- | --- | --- | --- | --- |
| (Intercept) | **169.38** | **1** | **39.13** | **< 0.001** |
| Subpopulation age | **0.04** | **1** | **0.01** | **0.923** |
| Subpopulation | **2866.02** | **7** | **94.59** | **< 0.001** |
| Subpopulation age:Subpopulation | **829.35** | **7** | **27.37** | **< 0.001** |
| Residuals | **653.63** | **151** | **NA** | **NA** |

*Table S5. Summary of the Linear Model (LM) examining the effect of subpopulation age and subpopulation on nest density. Shown are estimates, standard errors, the test statistic t, and the p values.*

| variable | Estimate | Std. Error | t value | p |
| --- | --- | --- | --- | --- |
| Intercept | **6.97** | **1.11** | **6.26** | **< 0.001** |
| Subpopulation age | **0** | **0.03** | **-0.1** | **0.923** |
| Subpopulation B | **21.21** | **1.32** | **16.09** | **< 0.001** |
| Subpopulation C | **-1.94** | **2.23** | **-0.87** | **0.386** |
| Subpopulation D | **-2.14** | **1.53** | **-1.4** | **0.164** |
| Subpopulation E | **30.95** | **2.05** | **15.12** | **< 0.001** |
| Subpopulation F | **4.03** | **6.86** | **0.59** | **0.558** |
| Subpopulation G | **3.04** | **3.63** | **0.84** | **0.404** |
| Subpopulation H | **5.17** | **2.15** | **2.4** | **0.017** |
| Subpopulation age:Subpopulation B | **-0.17** | **0.04** | **-3.91** | **< 0.001** |
| Subpopulation age:Subpopulation C | **-0.05** | **0.08** | **-0.6** | **0.55** |
| Subpopulation age:Subpopulation D | **0.28** | **0.06** | **5.06** | **< 0.001** |
| Subpopulation age:Subpopulation E | **-1.23** | **0.12** | **-10.48** | **< 0.001** |
| Subpopulation age:Subpopulation F | **-0.69** | **0.5** | **-1.38** | **0.17** |
| Subpopulation age:Subpopulation G | **0** | **0.32** | **0.01** | **0.993** |
| Subpopulation age:Subpopulation H | **-0.85** | **0.23** | **-3.68** | **< 0.001** |

*Figure S3. Density kernel of Nearest Neighbour Distance to previously occupied nests in the first year of occupancy.*

*Table S6. Parameter estimates for the Non-linear Least Squares Regression (NLSR) of Nearest Neighbour Distance (NND) against subpopulation age, where a is the initial NND (km) and b is the minimum NND (km).*

| Subpopulation | a | b |
| --- | --- | --- |
| A | 98.29 | 0.51 |
| B | 30.14 | -0.64 |
| C | 26.06 | 2.94 |
| D | 44.09 | 0.45 |
| E | 30.32 | -0.73 |
| F | 29.38 | 15.87 |
| G | 22.43 | 16.95 |
| H | 3.81 | 7.00 |

*Figure S4. Mean nearest neighbour distance to other occupied nests (oNND) against subpopulation size. Dots represent raw values, line represents fitted non-linear least square model.*

*Figure S5. Nearest neighbour distance to other occupied nests (oNND) against nest age. Dots and bars represent mean ± confidence intervals at each age, line represents fitted linear model with (shaded) confidence intervals.*
